# A Systematic Review on Assessing Assessments: Unveiling Psychometric Properties of Instruments for Reactive Attachment Disorder and Disinhibited Social Engagement Disorder in Minors under Protective Measures

**DOI:** 10.3390/children11020144

**Published:** 2024-01-24

**Authors:** Florencia Talmón-Knuser, Miriam Soler, Francisco González-Sala, Laura Lacomba-Trejo, Paula Samper-García

**Affiliations:** 1 Department of Developmental Psychology and Education, Faculty of Health Science, Catholic University of Uruguay, Montevideo 11600, Uruguay; florencia.talmonk@ucu.edu.uy; 2 Faculty of Psychology and Speech Therapy, Universitat de València, 46010 Valencia, Spain; misofuen@alumni.uv.es; 3 Department of Developmental and Educational Psychology, Faculty of Psychology and Speech Therapy, Universitat de València, 46010 Valencia, Spain; francisco.gonzalez-sala@uv.es (F.G.-S.); laura.lacomba@uv.es (L.L.-T.); 4 Department of Basic Psychology, Faculty of Psychology and Speech Therapy, Universitat de València, 46010 Valencia, Spain

**Keywords:** reactive attachment disorder, children, adolescents, psychometric proprieties, COSMIN, systematic review

## Abstract

Background: Reactive attachment disorder (RAD) and disinhibited social engagement disorder (DSED) manifest in individuals facing attachment system challenges, particularly observed in minors under protective measures. The lack of standardized tools for assessing these difficulties and uncertainty about the most effective instruments from a psychometric perspective prompted this study. Aim: Using the COSMIN checklist, we systematically reviewed instruments assessing RAD, adhering to PRISMA. Methodology: Examined tools included the Disturbance Attachment Interview, Preschool Age Psychiatric Assessment, Relationship Patterns Questionnaire, Assessment of RAD and DSED, Development and Well-Being Assessment, and Reactive Attachment Disorder Questionnaire. Results: Of the 10 articles analyzed, the results highlight a research emphasis on internal consistency and structural and construct validity, sidelining other properties. Conclusion: Most articles review structural validity and internal consistency. These measures are satisfactory but insufficiently evaluated. It is necessary to evaluate these tools using other indicators such as cross-cultural validity, measurement error, or responsiveness in adolescents under protective measures.

## 1. Introduction

The inclusion of attachment disorders in the DSM-III marked a significant milestone, encompassing manifestations of Reactive Attachment Disorder (RAD) and Disinhibited Social Engagement Disorder (DSED) [1]. In the DSM-5-TR, these challenges are categorized separately [2,3]. The key features of RAD in young children include (a) absence of attachment behaviors directed toward the primary caregiver, (b) failure to seek and respond to comfort in distressing situations, (c) diminished social and emotional reciprocity, and (d) disruptions in emotional regulation. Notable characteristics of DSED include (a) little caution in approaching unfamiliar adults; (b) willingness to be with strangers; (c) lack of appropriate social and physical boundaries, evidenced by overly close interaction with unfamiliar adults; and (d) seeking close physical contact [3].

Observing attachment disorders in children involves examining the shallowness and conflict in their relationships, stemming from a lack of trust due to negative experiences with primary caregivers. Children with RAD struggle to trust others, even when encountering kindness. In contrast, those with DSED exhibit indiscriminate trust, placing them at a higher risk of physical, sexual, and emotional harm [4].

RAD and DSED relate to a failure in the normative attachment system that a baby should develop with their primary caregiver, experiencing a lack of access to the protection and security the caregiver should provide [5]. Certain risk factors, particularly adverse childhood experiences, contribute to the development of RAD or DSED. These include psychological maltreatment, sexual abuse, neglect, parental alcoholism, familial drug use, caregiver mental health issues, or the absence of a consistent primary caregiver, potentially due to institutionalization, recurring protective measures changes, parental incarceration, or parental abandonment [6]. Younger children are more susceptible to negligent or abusive behaviors from caregivers, leading to situations of neglect and an increased risk of removal from their biological family and institutionalization [7].

Furthermore, DSED and RAD are frequently diagnosed in children who have been institutionalized, post-institutionalized, or placed in foster homes. These children often display self-soothing behaviors, discomfort in social interactions, and aggression toward peers [8]. Additionally, they commonly exhibit other associated personal, developmental, emotional, social, and behavioral difficulties [9,10]. Indeed, the experience of suffering from DSED or RAD is associated with a higher likelihood of presenting other emotional or behavioral difficulties [10] including anxiety, depression, emotional insecurity, attention-deficit/hyperactivity disorder, oppositional defiant disorder, a deficit in social skills, and an increased probability of being a victim or perpetrator of bullying [11,12,13,14,15,16,17,18,19]. DSED and RAD can also influence the way the child interacts with peers and caregivers, thereby complicating the child’s adaptation to the new family context. Difficulties associated with the inability to establish healthy emotional bonds with caregivers are a clear risk factor for the failure of protective measures such as foster care or adoption [20,21,22]. Additionally, institutionalization appears to be a risk factor for the development and persistence of DSED and RAD; even though an initial improvement in symptoms when transferred to a protective institution, this improvement tends to be temporary. Inhibitory behaviors remain stable, while disinhibited behaviors worsen with prolonged stay, suggesting that institutionalization can exacerbate attachment disorders, particularly disinhibited behaviors [20,23,24,25].

Taking the above into consideration, it becomes crucial to assess and identify the presence of attachment problems (DSED or RAD) in children under protective measures. Knowing about their presence can assist in implementing intervention measures within the family and protection system to reduce the likelihood of protective measures failing [20,21,22]. This approach could mitigate the health, social, and economic impact of the failure of protective measures, both on the children and their families, as well as on society at large.

Despite the relevance and prevalence of RAD and DSED, there are few instruments available to assess these mental health problems. It is important to note that with the introduction of the DSM-5, DSED, and RAD became independent disorders [26]. In this context, some tools are based on the DSM-IV-TR, such as The Child and Adolescent Psychiatric Assessment-RAD assessment (CAPA-RAD) [27], while others consider criteria from both the DSM-IV-TR and DSM-5, like the Disturbance of Attachment Interview [25]. In any case, it is necessary to identify tools based on the DSM-5 or DSM-5-TR [26]. Furthermore, most instruments focus on identifying disinhibited symptoms, as they are easier to observe than inhibited symptoms [28].

Lastly, there is still an ongoing debate about whether DSED and RAD symptoms are exclusive to childhood or can persist into adolescence and adulthood [17,29,30]. The difference between DSED and RAD in adolescence, as opposed to only in childhood, has not been conclusively established [25]. Therefore, the analysis of the psychometric properties of assessment instruments for DSED and RAD could shed light on these issues.

Given their significant relevance in the context of minors under protection, it is crucial to be aware of instruments that have demonstrated appropriate psychometric properties in these settings, as well as their quality. Identifying the strengths and weaknesses of these instruments can lay the groundwork for adapting these tools to other samples, contexts, languages, or countries and improving their psychometric quality.

### 1.1. Instruments Assessing Attachment Disorders

The characteristics of the most widely used instruments aimed at assessing RAD and DSED, which have been included in this study, are described below.

- Disturbance Attachment Interview (DAI): The DAI, developed by Smyke and Zeanah [31], is a semi-structured interview consisting of 12 items administered to a primary caregiver or someone well-acquainted with the child. It aims to assess signs related to disordered attachment and symptoms of both RAD and DSED. The items cover inhibited behaviors for RAD diagnosis, such as the absence of a preferred adult, lack of comfort-seeking, and limited social reciprocity. For DAI diagnosis, disinhibited behaviors are evaluated, including a lack of caution with strangers and a willingness to go with unknown adults. Scoring for the DAI ranges from 0 to 10 for RAD and 0 to 8 for DSED [32].
- The Preschool Age Psychiatric Assessment (PAPA): The authors of [33] developed the PAPA, a caregiver-reported questionnaire designed for preschoolers aged 2–8 years. This assessment evaluates RAD and DSED based on DSM-5 criteria. RAD classification requires meeting RAD criteria A1 and one or more criteria B. For DSED, participants must meet at least two DSED criteria [34].
- Relationship Patterns Questionnaire (RPQ): The RPQ, created by Kurth and Pokorny [35], employs a 10-item Likert scale to evaluate RAD symptoms, encompassing both RAD and DSED. Six items describe inhibited behaviors, while four describe disinhibited behaviors [36].
- Reactive Attachment Disorder and Disinhibited Social Engagement Disorder Assessment (RADA): Developed by Lehmann et al. [26], the RADA assessment follows DSM-5 criteria. It features 11 items for RAD and 9 for DSED. TAR includes two factors, incapacity to seek/accept comfort and low socioemotional responsiveness/emotional dysregulation, whereas DSED has one factor related to indiscriminate behaviors [26].
- Development and Well-Being Assessment RAD/DSED (DAWBA RAD/DSED): A section within the DAWBA interview [25] that includes 14 items derived from the Child and Adolescent Psychiatric Assessment for RAD/DSED. These items assess social behaviors of concern to caregivers, scoring from 0 to 10 for TAR and 0 to 18 for DSED [37].
- RAD Questionnaire (Questionnaire Disorder Attachment Reactive): The RAD Questionnaire, developed by Minnis et al. [38,39], consists of 17 items evaluating both reactive and disinhibited attachment disorders concurrently. Scores on this questionnaire range from 0 to 51, with higher scores indicating more severe attachment disorder symptoms [28].

### 1.2. The Current Study

The aim of the present study was to assess the psychometric properties of instruments evaluating attachment disorders in samples of minors under protective measures. To achieve this, a systematic review was conducted, adhering to PRISMA standards and utilizing the Consensus-based Standards for the selection of health status Measurement Instruments (COSMIN) checklist for systematic reviews of Patient-Reported Outcome Measures (PROMs) [40]. Information was gathered for each analyzed instrument, including authors, sample characteristics, country, design quality of the studies, and measurement properties as indicated in the studies.

## 2. Materials and Methods

### 2.1. Search Strategy

A systematic review of the scientific literature related to DSED and RAD was conducted according to the guidelines established by the PRISMA statement [40]. The search was conducted from January 2022 to March 2023 on the Web of Science. The names of the assessment instruments were combined using Boolean terms along with words related to the evaluation of psychometric properties and terms referring to the protective care system. The combination of these Boolean terms can be observed in Table 1.

### 2.2. Study Selection

This review included articles analyzing the psychometric properties of instruments assessing RAD and DSED in minors within the protective care system. To ensure data comprehensiveness, articles meeting the criteria described below were included: (1) Original articles published in English or Spanish, (2) published between 2000 and 2022, (3) with samples of minors in adoption or residential/foster care, and (4) addressing the study of psychometric properties of instruments evaluating RAD or DSED.

Exclusions comprised (1) gray literature (doctoral theses, conference communications, or press articles), (2) articles using questionnaires but lacking psychometric information, and (3) systematic reviews of articles.

### 2.3. Procedure

All records were screened in a blinded manner by two authors (FG-S and MS) and when there was disagreement, a third reviewer interceded (LL-T). After reviewing the title and abstract to identify articles that analyzed the psychometric properties of instruments, works that did not meet the inclusion criteria were excluded (*n* = 189).

**Figure 1:**
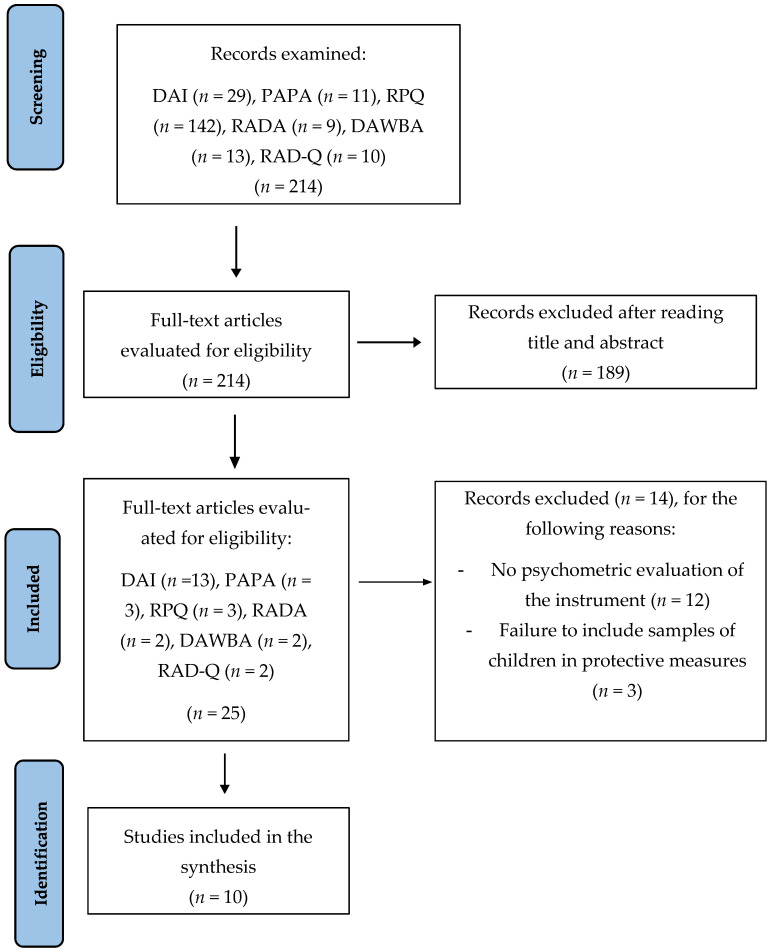
Flow of information through the different phases of a systematic review.

From the selected articles (*n* = 25), the full text was consulted, and once again, those that did not address the study of any instrument property were eliminated. According to this, twelve were eliminated because they did not evaluate psychometric properties, and three were eliminated because they did not refer to children in protective measures, resulting in a total of 10 articles. Among these, four focused on the DAI, one on the PAPA, two on the RPQ, one on the RADA, one on the DAWBA RAD/DSED, and two on the RAD Questionnaire. The entire process is outlined in Figure 1.

### 2.4. COSMIN Checklist for Systematic Reviews of PROMs

To thoroughly assess the methodological quality of the studies, the COSMIN checklist for systematic reviews of Patient-Reported Outcome Measures (PROMs) was utilized. This checklist distinguishes between “standards” and “criteria”, with the former referring to the requirements set by each research study, indicating the quality of the study itself. Meanwhile, the latter, the “criteria”, pertain to what would constitute a good measurement (the quality of the PROM) [40]. The objective of this checklist is to evaluate the methodological quality of assessment instruments.

The checklist is divided into three parts and 10 boxes. The first part includes contributions from procedures aimed at conducting a systematic review according to guidelines such as PRISMA [41]. The second part encompasses the criteria used to assess the measurement quality of PROM instruments. The third and final part corresponds to the evaluation of the interpretability and feasibility of the PROM, as well as the formulation and reporting of the systematic review. For the optimal use of the COSMIN guide, the authors recommend employing the checklist as a modular tool, filling in the relevant boxes [40].

## 3. Results

In the following section, the psychometric properties of each of the instruments selected for this study will be presented in detail.

### 3.1. Characteristics of the Samples of the Studies Analyzed

Four articles analyzed the psychometric properties of the Disturbance Attachment Interview (DAI). These works included samples of between 55 and 853 people, all of whom were children aged between 4 months [32] and 15 years [15] who were in protective measures. On the other hand, only one work analyzed the psychometric properties of The Preschool Age Psychiatric Assessment (PAPA) [33]. It had a sample of 400 adolescent and young adult participants of both sexes from residential centers. In this case, although the instrument is for younger children, it was applied to adolescents.

On the other hand, an article psychometrically analyzed the Relationship Patterns Questionnaire (RPQ). It had a sample of 135 children with an average age of around 7 years. This work compared normal population, foster children, and hospitalized children [42]. The Reactive Attachment Disorder and Disinhibited Social Engagement Disorder Assessment (RADA), on the other hand, had one study that analyzed its properties with a sample of over 300 children aged between 11 and 18 in foster care [26].

The Development and Well-Being Assessment RAD/DSED (DAWBA) was evaluated in a study that included 122 children adopted from birth to 10 years of age [12]. Finally, the Questionnaire Disorder Attachment Reactive (RAD-Questionnaire) is analyzed in two studies with samples of 55 children [28] and 182 children [39] in protective situations, between 12 months [28] and 16 years [39], all of whom had experienced various changes within the foster care resource, both residential and family. More detailed information on these aspects can be found in Table 2.

### 3.2. Methodological and Measurement Quality of the Instruments Results

In general, none of the studies assessing the psychometric properties of the instruments include all eight pertinent items as outlined in the COSMIN Guide. Moreover, none of the manuscripts provide indices for cross-cultural validity, establish relationships or comparisons with the gold standard, or address responsiveness. The methodological quality of the selected studies is summarized in Table 3.

- Disturbance Attachment Interview (DAI): In the majority of instances, the primary focus lies on structural validity and internal consistency. In this context, two out of the four studies conducted analyses on structural validity, both employing confirmatory factor analysis. However, one of them [43] had a limited sample size (<5 times the number of items). Regarding criterion validity, only one study examined it [43]. Concerning hypothesis testing and construct validity, several tests were conducted, but without establishing a priori hypotheses.

In terms of reliability (Test–Retest) and measurement error, only one study provided information [43]. Concerning consistency, two out of the four studies estimated Cronbach’s alpha and inter-rater reliability [28,32]. Both studies achieved satisfactory inter-rater reliability. Regarding the internal consistency of the instrument, Smyke et al. [32], the authors who developed the interview, obtained satisfactory internal consistency values.

In the case of the study by Kliewer-Neuman et al. [28], it was deemed unacceptable even after relevant statistical modifications. Thus, after removing item 5, an α = 0.72 was obtained on the DSED subscale, and the secure base disturbance scale, α = 0.42, was obtained, which was deemed insufficient. This study also assessed consistency over time with weighted kappa, although it was only performed in 30% of the interviews, yielding substantial results (0.61 to 0.80) [44]. Sufficient data were also provided to calculate the Limits of Agreement (LoA) for measurement error and information on concurrent validity, considering the relationship between DAI and the SDQ questionnaire that assesses emotional, behavioral, and hyperactive symptoms [43].

The study by Kliewer-Neumann et al. [28] evaluated the association between DAI and RAD, demonstrating concurrent validity with the inhibition subscale of DAI but not with disinhibition. In the case of the disinhibition scale, an association was found with tests related to the strange situation.

No study assessed the transcultural validity of the instruments, nor responsiveness (Area Under the Curve), and there were not enough studies testing hypotheses to calculate the percentage of agreement as established in the COSMIN criteria.

- The Preschool Age Psychiatric Assessment (PAPA): A study conducted by Seim et al. [33] evaluated the psychometric properties of the PAPA, with a primary focus on criterion validity, measurement error, and discriminant validity. The study produced positive results concerning the two-factor factorial structure (inhibited and disinhibited) as well as discriminant validity. It was observed that RAD and DSED were distinct constructs from each other and other mental health issues.
- Relationship Patterns Questionnaire (RPQ): A study conducted by Schöder et al. [42] examined the internal consistency and measurement error of the RPQ. The study reported satisfactory values for internal consistency (overall scale α = 0.82; inhibition subscale α = 0.75; disinhibition subscale α = 0.81). Regarding criterion validity analysis and responsiveness, calculations were undertaken in the study by Schröder et al. [15]. Significant Area Under the Curve (AUC) values were obtained, signifying diagnostic accuracy, with lower accuracy observed in boys compared to girls. The study also proposed diagnostic cut-off points.
- Reactive Attachment Disorder and Deshinhibited Social Engagement Disorder Assessment (RADA): A singular study evaluated the psychometric properties of the RADA [26]. The study centered on analyzing its factorial structure by suggesting a three-construct factorial solution (DSED: indiscriminate behaviors with strangers; RAD1: inability to seek/accept comfort; RAD2: withdrawal/hypervigilance). Additionally, the study explored internal consistency, as well as criterion and construct validity.
- Developmental and Well-Being Assessment RAD/DSED (DAWBA RAD/DSED): Lehmann et al. [12] examined structural validity through a confirmatory factor analysis with two factors, aligning with the DSM-5 definition. Concerning construct validity, the study sought to differentiate difficulties between SDQ, DSED, and RAD.
- RAD Questionnaire: Minnis et al. [39] evaluated structural validity, internal consistency, temporal consistency, measurement error, and criterion validity. Adequate indicators were obtained in all cases. Consequently, the tool demonstrated satisfactory internal consistency for use in research settings (α = 0.7). The relationship between the questionnaire and the SDQ was examined, revealing very high correlations, which, in general terms, may not necessarily be positive.

On the other hand, the study by Kliewer-Neumann et al. [28] solely conducted hypothesis testing for construct validity without formulating an a priori hypothesis. Moderate relationships were observed between the RAD Questionnaire and the DAI.

### 3.3. Criteria Measurement: Quality of the Instruments

The quality of the assessment measures was evaluated by taking into account the updated criteria for good measurement properties outlined in the COSMIN manual [40] (Table 4).

### 3.4. Strength of Evidence

Referring to Table 5, it can be observed that none of the studies provided information for all nine criteria in the COSMIN checklist. According to the modified GRADE criteria [45], most instruments exhibited low to moderate evidence.

## 4. Discussion

The main objective of this systematic review was to evaluate the psychometric properties of assessment instruments for Reactive Attachment Disorder (RAD) and Disinhibited Social Engagement Disorder (DSED) in minors at risk through a systematic review following the COSMIN checklist [40]. As a primary finding, we note that the general trend in the examined studies is to report on internal consistency and structural validity. Additionally, the majority conducted hypothesis testing for construct validity, but without a priori hypotheses. Overall, there is a lack of evidence regarding reliability, cross-cultural validity, measurement error, and responsiveness. Therefore, we conducted an exhaustive analysis of the instruments, though it may be limited due to the nature of the studies.

To date, this is the first study evaluating the psychometric properties of instruments assessing RAD and DSED in at-risk minors. Consequently, our results are challenging to compare with previous works. However, the study by Wright et al. [46], which conducted a systematic review and meta-analysis on instruments assessing and/or diagnosing attachment problems, concluded that assessments of the psychometric properties of these instruments often neglect cross-cultural validity or longitudinal reliability. Therefore, more high-quality scientific research is needed to examine the validity of available instruments and provide sufficient evidence for their consideration in clinical diagnosis.

As far as we know, the reliability indices of different instruments, while mostly adequate (alpha between 0.70 and 0.82), are not sufficient to use the tools as diagnostic instruments for RAD/DSED. Most studies support the two-factor RAD and DSED factorial structure, but some authors suggest the existence of three factors (DSED: indiscriminate behaviors with strangers; RAD1: inability to seek/accept comfort; RAD2: withdrawal/hypervigilance) [26], in line with the proposals of the DSM-5-TR and opening avenues for further research into a potential new diagnostic category associated with RAD. However, much more evidence and research are needed in this regard.

Furthermore, studies must conduct cross-cultural investigations testing the functioning of instruments in different countries. Despite studies being conducted in various countries such as Germany [28,42,43], Norway [12,26,33], Scotland [39], Romania [32], and Finland [15], there is a lack of evidence for cross-cultural construct validity through multigroup structural equation modeling. Additionally, some studies focus on assessment interviews for these disorders, providing only evidence of inter-rater reliability [28]. Therefore, future research should assess internal consistency over time, content and construct validity, cross-cultural validity, and the structure, efficacy, and efficiency of interviews. Similarly, we identified a scarcity of instruments in Spanish, pointing towards a potential avenue for future exploration. The adaptation and validation of the psychometric properties of these instruments in Spanish-speaking countries could prove crucial for their application in clinical and social contexts.

Future studies should encompass samples from diverse countries and consider analyzing psychometric properties with additional indices. For instance, conducting exploratory factor analyses could be insightful, especially considering the ongoing debate surrounding these instruments (two single factors or two factors with subfactors). Likewise, these investigations could contribute to addressing inquiries about the prevalence of attachment problems in adolescents, shedding light on their persistence throughout the life cycle.

Despite the significant contributions of this work, evaluating key RAD/DSED detection measures in at-risk or protective minors, it is essential to acknowledge limitations. Strict selection criteria may have limited the scope of the review, as only works published in Spanish and English were considered. The COSMIN checklist [40], while enhancing the structure and quality of our work, leaves room for subjectivity in data interpretation and quality assessment. Future research should replicate the search in other databases to provide additional information. For example, in criteria where evaluation is based on a time period, it depends on the construct being assessed and what the researcher deems appropriate. Furthermore, even with adequate reliability, a high Cronbach’s alpha does not guarantee that the desired construct is being measured or that no essential concepts are missing. Similarly, having high test–retest reliability or responsiveness does not imply that all items are important or that central concepts have not been overlooked. This highlights the possibility of assessing an incomplete or incorrect construct reliably when following the checklist.

On the other hand, research on reactive attachment disorder is limited, resulting in few studies delving into its assessment and even fewer evaluating the psychometric properties of assessment instruments. This limitation is particularly pronounced when considering studies focused on minors at risk or under protection, who, as we know, are more susceptible to experiencing RAD/DSED than minors outside the protection system [9]. Finally, it is important to note that the search was conducted solely in one database, the one that aggregates a greater number of impactful publications. Nevertheless, future studies should replicate this search in other databases to contribute additional information on the subject.

However, this work contributes to expanding knowledge about assessment instruments for reactive attachment disorder in the context of child protection, laying the groundwork for future assessments of the psychometric properties of these instruments. Having identified areas for improvement in assessing the psychometric properties of these instruments, future studies could be designed with a cross-cultural and longitudinal approach to gathering more scientific evidence in line with COSMIN checklist criteria, including measurement error, cross-cultural validity, test–retest reliability, responsiveness, criterion validity, and construct validity [40]. In this way, progress can be made in understanding, assessing, and treating RAD/DSED. Our research endeavors to enhance the clinical practices of professionals by offering insights into the appropriateness and characteristics of RAD/DSED assessment instruments. Given the association of RAD or DSED with a higher prevalence of mental health issues in children [10,12,15,17,22,47], especially in children where protective measures have been taken at a younger age [14,48], there is a compelling rationale for implementing RAD/DSED assessments within the realm of child protection.

In conclusion, we note that this is the first study to examine and assess the psychometric properties of six instruments that assess RAD/DSED in a sample of adopted or foster children and adolescents. Regarding the evaluated measures, it can be concluded that all of them exhibit good structural validity, adequate internal consistency, and generally positive results concerning measurement error.

## Figures and Tables

**Table 1:** Keywords used for the Boolean search.

Questionnaires		Psychometric Properties		Samples
Disturbance Attachment Interview OR DAI The Preschool Age Psychiatric Assessment OR PAPA Relationship Patterns Questionnaire OR RPQ Reactive Attachment Disorder and Disinhibited Social Engagement Disorder Assessment OR RADA Developmental and Well-Being Assessment RAD/DSED OR DAWBA RAD/DSED Questionnaire Disorder Attachment Reactive OR RAD Questionnaire	AND	validity OR measurement error OR reliability OR invariance OR cross OR retest OR consistency OR responsive	AND	foster OR adopt OR residential foster care

**Table 2:** Characteristics of samples used in published scientific articles on psychometric properties of questionnaires assessing RAD and DSED.

Article and Instrument	Application Information (Procedure)	Country, Sample, and Characteristics of Samples
Kliewer-Neumann et al. (2018)—DAI and RAD [28]	Audio-recorded interviews with parents and observation of the child in the laboratory. Time 30 min. Two trained evaluators independently analyzed the interviews.	Germany. *n* = 55. Foster children from German youth welfare programs. The children ranged in age from 12 to 82 months (M = 35.87; SD = 18.37), with 50.9% being female (*n* = 28). These children had been in foster care for an average of 78 days, and some of them had experienced up to 5 changes in placement or families.
Kliewer-Neumann et al. (2015)—DAI [43]	Two observations are made of the caregiver–child interaction. In the home, the Stranger at the Door procedure and questionnaires are administered. Door procedure and questionnaires are administered. It is videotaped for 3 h. In the laboratory, the Stranger at the Door procedure and the IAD are administered. Door procedure and the DAI.	Germany. *n* = 50. Foster children aged between 34 and 104 months (M = 68.32; SD = 19.29), with 48% being female (*n* = 24). The children had been living with their foster families for an average of 45.36 months.
Smyke et al. (2002)—DAI [32]	Interview educators in the foster care institution and foster parents at home. In addition, files of institutionalized children are reviewed. Interviews are conducted in Romanian and English with the help of an interpreter. The duration of the assessment is not specified. Foster mothers receive a compensation of USD 10.	Romania. *n* = 94. Children residing in a large institution in Bucharest (*n* = 32), young children residing in the same institution but in a pilot unit (*n* = 29), and young children living in foster care who had never been institutionalized (*n* = 33). All children ranged in age from 4 months to 68 months.
Elovainio et al. (2015)—DAI [15]	Self-report questionnaires are administered to children and adoptive parents. The duration of the assessment is not specified.	Finland. *n*= 853. Boys and girls adopted as part of a Finnish adoption study (FinAdo), involving international adoption. The participants’ ages ranged from 6 to 15 years (M = 8.5; SD = 2.9), and they had been adopted for an average of 2.4 years (SD = 1.3). Prior to adoption, they had experienced various placement resources, including foster care and residential care, among others. An adapted version of the DAI was administered.
Seim et al. (2020)—PAPA [33]	Psychiatric interviews are conducted with the adolescents and their primary educators in the residential centers. Four trained and experienced researchers conduct the assessment. Data are collected between June 2011 and July 2014. The duration of the assessment is not specified.	Norway. *n* = 400. Adolescents aged between 12 and 23 years, residing in Norwegian residential centers. The participants had a mean age of M = 16.7 (SD = 3.9), an average of 3.3 out-of-home placements (SD = 2.4), and the mean age of their first out-of-home placement was 12.5 years (SD = 3.9).
Schröder et al. (2019)—RPQ [42]	Children and parents are informed of the purpose of the study. Children’s and parents’ assessments are videotaped and transcribed. Evaluators are trained and certified to carry out the assessment of the instruments. Time of application and number of sessions are not specified.	Switzerland and Germany. *n* = 135. Children with a mean age of 7.17 years (SD = 1.40). The sample divided participants into three groups: general population (*n* = 34), with a mean age of 6.36 years (SD = 1.06); hospitalized and outpatient patients (*n* = 69), with a mean age of 7.39 years (SD = 1.42); and the foster care group (*n* = 32), with a mean age of 7.52 years (SD = 1.42). Children with an IQ ≤ 70 or with ASD were excluded.
Lehmann et al. (2020)—RADA [26]	The assessment takes place between October 2016 and March 2017. Foster parents and adolescents complete the instruments online or by telephone. Teenagers receive a gift card of 33 USD.	Norway. *n* = 320. Youth living in foster care for an average of 6.6 years (SD = 4.3), aged between 11 and 17 years (M = 14.8, SD = 2.0), with 56.8% being boys.
Lehmann et al. (2016)—DAWBA [12]	Data are collected between September 2011 and February 2012. Each child’s social worker completes The Child Protection Questionnaire. Approximate time between 10 and 20 min. Foster parents complete the questionnaires online. First the SDQ (approximately 10 min) and then the DAWBA RAD/DSED (approximately 5 min). Subsequently, they complete The Child Protection Questionnaire.	Norway. *n* = 122. Adopted children aged between 0 and 10 years in Norway, of which 57% were girls. Children with severe neurological problems are not included in the study.
Minnis et al. (2002)—RAD Questionnaire [39]	The RAD is filled in by the foster parents independently. Between 3 and 5 weeks later, they fill in the RAD again. Social workers provide information regarding the child protection file.	Scotland. *n* = 182. Scottish children residing in foster homes aged between five and sixteen years (M = 11) and had spent an average of 2.5 years with their current foster caregivers.

**Table 3:** Psychometric properties and methodological quality of the instruments according to the COSMIN guidelines.

Psychometric Property	Articles	Psychometric Property	Articles
*Structural validity*		*Measurement error*	
Excellent	4, 5, 7, 8, 10	Excellent	
Good		Good	
Fair		Fair	
Poor	2	Poor	
Unknown/NA	1, 3, 10	Unknown/NA	1, 2, 3, 4, 5, 6, 7, 8, 9, 10
*Internal consistency*		*Criterion validity*	
Excellent	2, 3, 6, 7, 10	Excellent	2, 4, 5, 6, 7, 10
Good		Good	
Fair		Fair	
Poor	5	Poor	
Unknown/NA	[12,15,28]	Unknown/NA	1,3,8
*Cross-cultural validity*/ *measurement invariance*		*Hypothesis testing for construct validity*	
Excellent		Excellent	1
Good		Good	9
Fair		Fair	
Poor		Poor	
Unknown/NA	1, 2, 3, 4, 5, 6, 7, 8, 9, 10	Unknown/NA	1,2,3,4,5,6,7,8,9,10
*Reliability*		*Responsiveness*	
Excellent	1, 3, 10	Excellent	
Good		Good	
Fair		Fair	
Poor		Poor	
Unknown/NA	1, 3, 4, 5, 6, 7, 8, 9	Unknown/NA	1, 2, 3, 4, 5, 6, 7, 8, 9, 10

Note: DAI: (1) Kliewer-Neumann, et al. (2018) [28]; (2) Kliewer-Neumann, et al. (2015) [43]; (3) Smyke et al. (2002) [32]; (4) Elovaino et al. (2015) [15]; PAPA (5) Seim et al. (2020) [33]; RPQ: (6) Schröder et al. (2019) [42]; RADA: (7) Lehmann et al. (2020) [26]; DAWBA: (8) Lehmann et al. (2016) [12]; RAD Questionnaire: (9) Kliewer-Neumann et al. (2018) [28]; (10) Minnis et al. (2002) [39]. NA: Not applicable.

**Table 4:** COSMIN results of the criteria of measurement (quality of the PROM).

Study and Instrument	Structural Validity	Internal Consistency	Crosscultural Validity Measurement Invariance	Reliability	Measurement Error	Criterion Validity	Hypothesis Testing for Content Validity	Responsiveness
Kliewer-Neumann et al. (2018)—DAI [28]	?	?	?	+	?	?	+	?
Kliewer-Neumann et al. (2015)—DAI [43]	?	+	−	?	?	+	?	?
Smyke et al. (2002)—DAI [32]	?	+	?	+	?	?	?	?
Elovainio et al. (2015)—DAI [15]	+	?	?	?	?	+	?	?
Seim et al. (2020)—PAPA [33]	+	+	?	?	?	+	?	?
Schröder et al. (2019)—RPQ [42]		+	?	?	?	+	?	?
Lehhmann et al. (2020)—RADA [26]	+	+	?	?	?	+	?	?
Lehmann et al. (2016)—DAWBA [12]	+	?	?	?	?	?	?	?
Kliewer-Neumann et al. (2018)—RAD [28]	?	?	?	?	?	?	+	?
Minnis et al. (2002)—RAD [39]	+	?	?	?	?	+	?	?

Note: 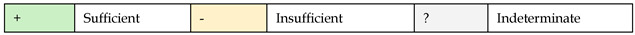.

**Table 5:** Strength of evidence for each study.

Study and Instrument	Structural Validity	Internal Consistency	Crosscultural Validity/ Measurement Invariance	Reliability	Measurement Error	Criterion Validity	Hypothesis Testing for Content Validity	Responsi Veness	% Strong- Moderate Evidence	Average Percentage Evidence of Instruments
Kliewer-Neumann et al. (2018)—DAI [28]	U	U	U	S	U	U	S	U	25%	28.13%
Kliewer Neumann et al. (2015)—DAI [43]	U	S	M	U	U	S	U	U	37.5%	
Smyke et al. (2002)—DAI [32]	U	S	U	S	U	U	U	U	25%	
Elovainio et al. (2015)—DAI [15]	S	U	U	U	U	S	U	U	25%	
Seim et al. (2019)—PAPA [33]	S	S	U	U	U	S	U	U	37.5%	37.5%
Schröder et al. (2019)—RPQ [42]	U	S	U	U	U	S	U	U	25%	25%
Lehmann et al. (2020)—RADA [26]	S	S	U	U	U	S	U	U	37.5%	37.5%
Lehmann et al. (2016)—DAWBA [12]	S	U	U	U	U	U	U	U	12.5%	12.5%
Kliewer-Neumann et al. (2018)—RAD [28]	U	U	U	U	U	U	S	U	12.5%	18.7%
Minnis et al. (2002)—RAD [39]	S	U	U	U	U	S	U	U	25%	
% strong-moderate evidence	40%	50%	0%	20%	0%	60%	20%	0%		
% limited conflicting evidence	0%	0%	0%	80%	0%	0%	0%	0%		
% unknown evidence	50%	50%	90%	80%	100%	40%	80%	100%		

Note: The right-hand column represents the % of strong-moderate evidence for each article, bottom row indicates the strength of evidence for each psychometric characteristic evaluated by COSMIN. 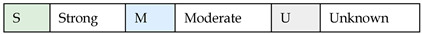.

## Data Availability

Not applicable.
